# Dataset of video game-based assessments in digital culture courses at Indoamerica University

**DOI:** 10.1016/j.dib.2024.111217

**Published:** 2024-12-09

**Authors:** Miguel Cobos

**Affiliations:** Facultad de Educación, Universidad Indoamérica, Quito, Ecuador

**Keywords:** Educational assessment, Gamification, Digital culture, Student performance, Learning analytics

## Abstract

This dataset contains evaluation results from video game-based assessments administered to first-level university students across six different academic programs at Universidad Indoamérica from October 2022 to August 2024. The data were collected using an adapted version of Pacman through the ClassTools.net platform, where traditional quiz questions were integrated into gameplay mechanics. The dataset comprises 1418 assessment attempts from students in Law, Medicine, Psychology, Clinical Psychology, Architecture, and Nursing programs, documenting their performance in digital culture and computing courses. Each record includes attempt number, timestamp, student identifier, gender, academic period, section, career program, and score achieved. The dataset enables analysis of student performance patterns, learning progression through multiple attempts, and comparative studies across different academic programs and periods. This information can support research in educational gamification, assessment design, and digital learning strategies in higher education.

Specifications Table

**Table:** 

Subject	Social Sciences: Education.
Specific subject area	Educational Technology and Assessment in Higher Education through Game-Based Learning in Computing and Digital Culture courses.
Type of data	Table.
Data collection	Data were collected through the ClassTools.net platform using an adapted Pacman video game for assessment. Questions about computing and digital culture were integrated into the game mechanics. All evaluations were conducted in computer laboratories, where students had a two-hour time limit with unlimited attempts allowed. Each attempt was automatically recorded with timestamp, student information, and score achieved.
Data source location	The data were collected at the Universidad Indoamerica, Machala y Sabanilla Postal Code: 170,301, Quito, Ecuador.
Data accessibility	Repository name: Mendeley Data Data identification number: 10.17632/vvx3p7ph59.5 Direct URL to data: https://data.mendeley.com/datasets/vvx3p7ph59/5
Related research article	none.

## 1 Value of the Data

• The dataset provides raw assessment records from gamified evaluations conducted in higher education across six distinct academic programs (Law, Medicine, Psychology, Clinical Psychology, Architecture, and Nursing), collected from first-level students in computing and digital culture courses [1]. This dataset stands out due to its integration of gamification mechanics within the assessment process. These records offer a unique opportunity for researchers to study how gamification fosters active participation, enhances engagement, and supports autonomous learning in diverse disciplines [2]. Additionally, it highlights the potential of integrating classic games, such as Pac-Man, into assessment tools through platforms like ClassTools.net, blending educational content with entertainment.
• The dataset includes 1418 assessment attempts from 353 unique students across four academic periods (October 2022 to August 2024). Each attempt is accompanied by detailed variables such as timestamps, performance scores, academic programs, gender, and repeated attempts within a two-hour evaluation window. This level of granularity enables researchers to explore patterns of performance improvement, persistence trends, and the motivational impact of gamification strategies like leaderboards and point systems.
• Researchers can reuse this dataset to conduct cross-disciplinary analyses and explore trends in gamified assessments across different academic programs. The consistent methodology for data collection through ClassTools.net allows for replication in other contexts, supporting comparative studies, predictive modelling, and longitudinal research in student performance.
• The dataset facilitates longitudinal analyses to identify learning patterns and inform Learning Analytics or educational data mining. These approaches promote collaboration between educators and institutions by providing actionable insights into student outcomes [3]. By documenting not only performance but also engagement through gamification, this dataset supports future research in data-driven education and gamification strategies.
• The dataset's detailed variables (e.g., timestamps, attempts, gender, and program) enable various research opportunities, such as analysing performance patterns across multiple attempts, examining gender-based differences in gamified assessments, and comparing outcomes between academic disciplines. This flexibility makes the dataset a valuable resource for researchers in education and gamification.
• The comprehensive data structure supports studies on the effectiveness of gamification in higher education assessments. Researchers can develop predictive models for student performance, evaluate the scalability of gamified tools in educational settings, and design evidence-based strategies for improving technology-enhanced learning.

## 2 Background

The integration of video games in educational assessment emerged from the need to transform traditional evaluation methods into higher education to promote student learning [4]. Pac-Man, with its proven effectiveness in higher education over two decades [5,6], has been successfully used to enhance student engagement and understanding in various academic contexts [7]. This dataset was compiled to document an alternative assessment approach that aims to reduce student stress and anxiety typically associated with conventional examinations. The strategy implemented at Universidad Indoamerica utilized the ClassTools.net platform to adapt the Pacman video game into an evaluation tool for computing and digital culture courses.

In these times where artificial intelligence can completely override evaluation, we are looking for ways to have effective tools to evaluate [8], transforming summative evaluation into formative evaluation. The methodology allowed students multiple attempts within a two-hour examination period, creating a less stressful and engaging learning environment [9].

The data collection spans four consecutive academic periods, documenting student interactions with this alternative assessment method across different academic programs, with each attempt automatically recorded under standardized conditions.

## 3 Data Description

The dataset is provided in a single Microsoft Excel file containing 1418 records of assessment attempts. Each record includes the following fields:

The **Attempt** field shows a sequential number indicating each student's attempt at the assessment. The **Date** field captures the timestamp of the assessment attempt in format DD/MM/YYYY HH:MM:SS. The **Student** field contains unique anonymized identifiers for each student (e.g., ‘Student1’, ‘Student2’).”, while **Sex** indicates the student's gender (Male/Female).

The **Academic period** field identifies four consecutive terms: QB22 (October 2022-February 2023), QA23 (April-August 2023), QB23 (October 2023-February 2024), and QA24 (April-August 2024). The **Section** field refers to the parallel within each academic program, created based on the number of enrolled students (1 or 2). The **Career** field identifies the academic program to which each student belongs (e.g., Medicine, Law, Psychology). While the course names may vary depending on the academic program, the content and assessments, including question banks, are identical across all careers. This ensures consistency in the delivery and evaluation of computing and digital culture courses, which are part of the foundational curriculum for all students. The **Score** field contains the numerical score achieved in each attempt. Students were instructed to reach 20,000 points to obtain the maximum grade (10/10), with scores scaled proportionally (score/2000) to determine the final grade.

The assessment used a bank of 120 questions randomly selected during each attempt. The variation in the number of attempts per student and score fluctuations between attempts are recorded in the dataset.

Each row represents a single assessment attempt, and all fields are consistently formatted across the dataset. Table 1, Table 2 show these details.

**Table 1:** Distribution of assessment attempts by academic period and program.

Academic Period	Total Students	Total Records	Programs
QB22 (Oct 22-Feb 23)	124	481	Law (2 sections), Medicine (2 sections), Psychology (1 section)
QA23 (Apr-Aug 23)	52	192	Psychology, Clinical Psychology, Medicine
QB23 (Oct 23-Feb 24)	73	348	Law, Psychology, Clinical Psychology
QA24 (Apr-Aug 24)	104	397	Law, Architecture, Clinical Psychology, Medicine, Nursing

**Table 2:** Assessment records summary.

Field	Description	Range/Values
Attempt	Number of attempts per student	1 to maximum attempts observed
Score	Points obtained in game	0 to maximum score observed
Section	Class group	1, 2
Gender	Student gender	Male, Female

## 4 Experimental Design, Materials and Methods

The experimental design followed established principles for integrating video games into educational assessment, supporting the methodology through game mechanics proven in various academic contexts. According to a recent analysis of game-based assessment studies [7], this approach enhances both student engagement and academic performance. These findings reinforce the validity of integrating gamified mechanics into assessments as an effective way to foster meaningful learning.

### 4.1 Assessment platform setup

**Fig. 1:**
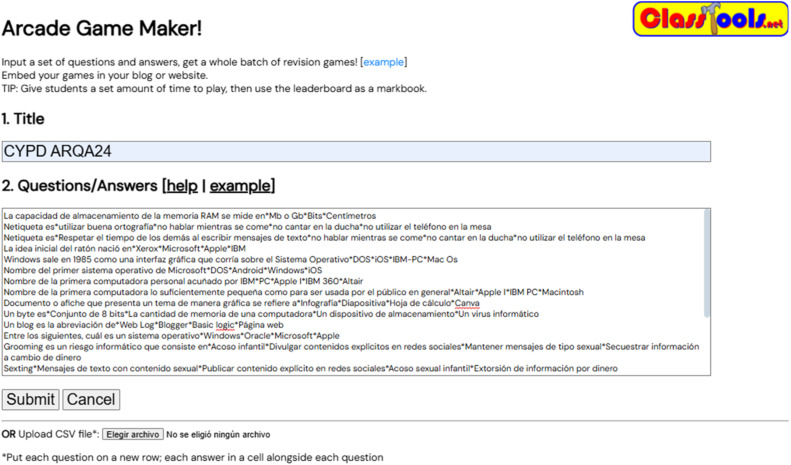
Question bank input system interface at Classtools.net.

The ClassTools.net Arcade Game Generator platform was used to create a Pacman-based assessment. A question bank of 120 items about computing and digital culture was integrated into the game mechanics through the platform's interface, see Fig. 1. Questions covered course content about the origin of communication, early computers, devices, and digital technological advances in society.

In this way, questions are entered to integrate them into the game mechanics. Each question should be placed on a fresh line, with the following structure:

Question A*correct answer*incorrect answer*incorrect answer 2*incorrect answer 3

### 4.2 Game mechanics and assessment design

• Initial phase: Students must correctly answer a random question from the question bank to start the game
• Main gameplay:
  ○ Players earn 5 points for each dot collected in the Pacman maze
  ○ Traditional power-ups in Pacman were replaced with fruits distributed throughout the board
  ○ After collecting a fruit, ghosts turn blue and can be pursued for a limited time
  ○ Catching a blue ghost awards 50 points
• Life and Question System:
  ○ Players start with one life
  ○ Collision with a non-blue ghost results in loss of life
  ○ To regain life and continue playing, players must answer random questions from the question bank
  ○ Each subsequent life loss increases the number of questions that must be answered correctly
  ○ Correct answers award 200 points per question and restore the life
  ○ Any incorrect answer ends the game immediately
  ○ This progressive difficulty system creates both a challenge and an opportunity to earn more points

**Fig. 2:**
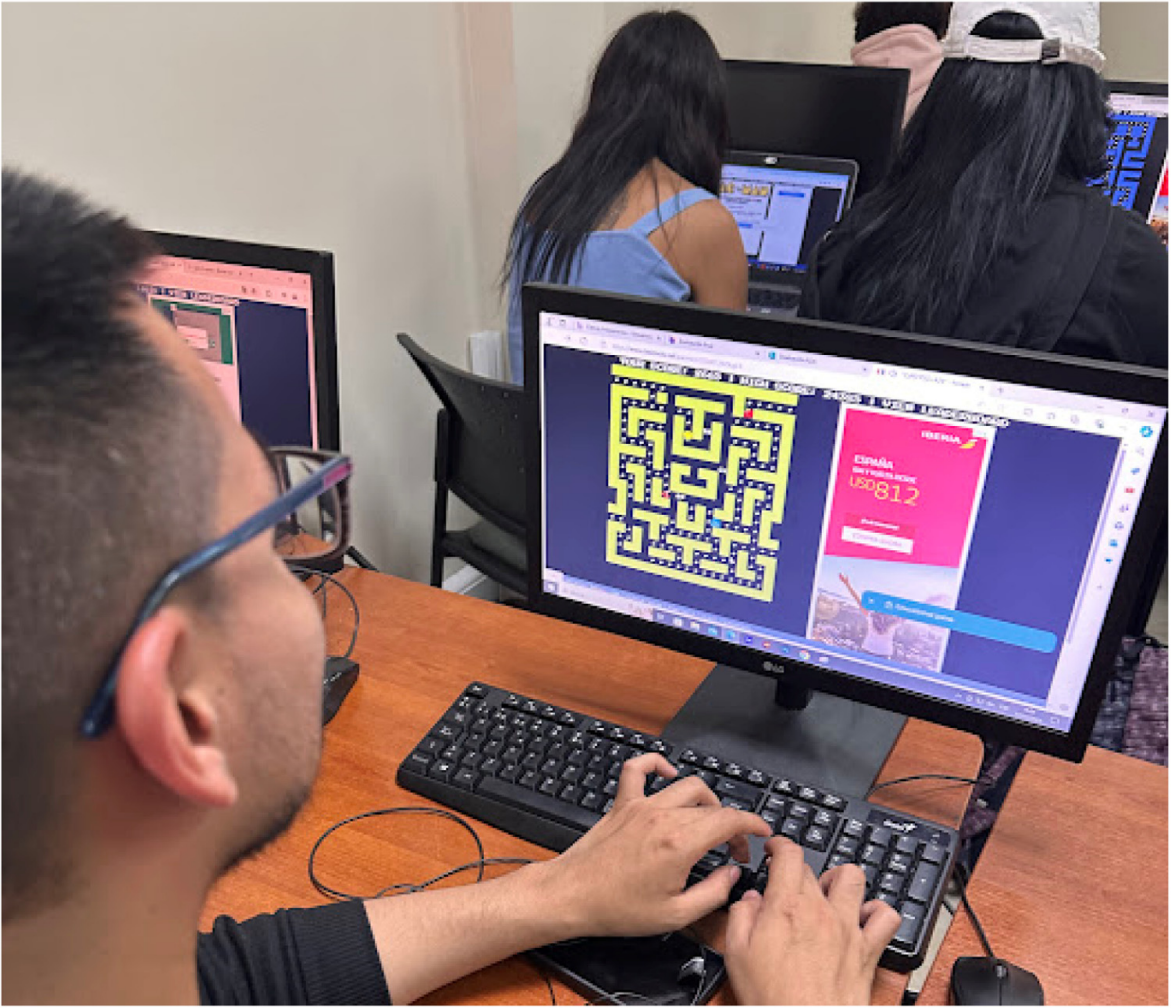
Student engaging with the Pacman game during assessment.

Fig. 2. shows students engaged with the game, showing the game interface and maze navigation.

### 4.3 Implementation process

**Fig. 3:**
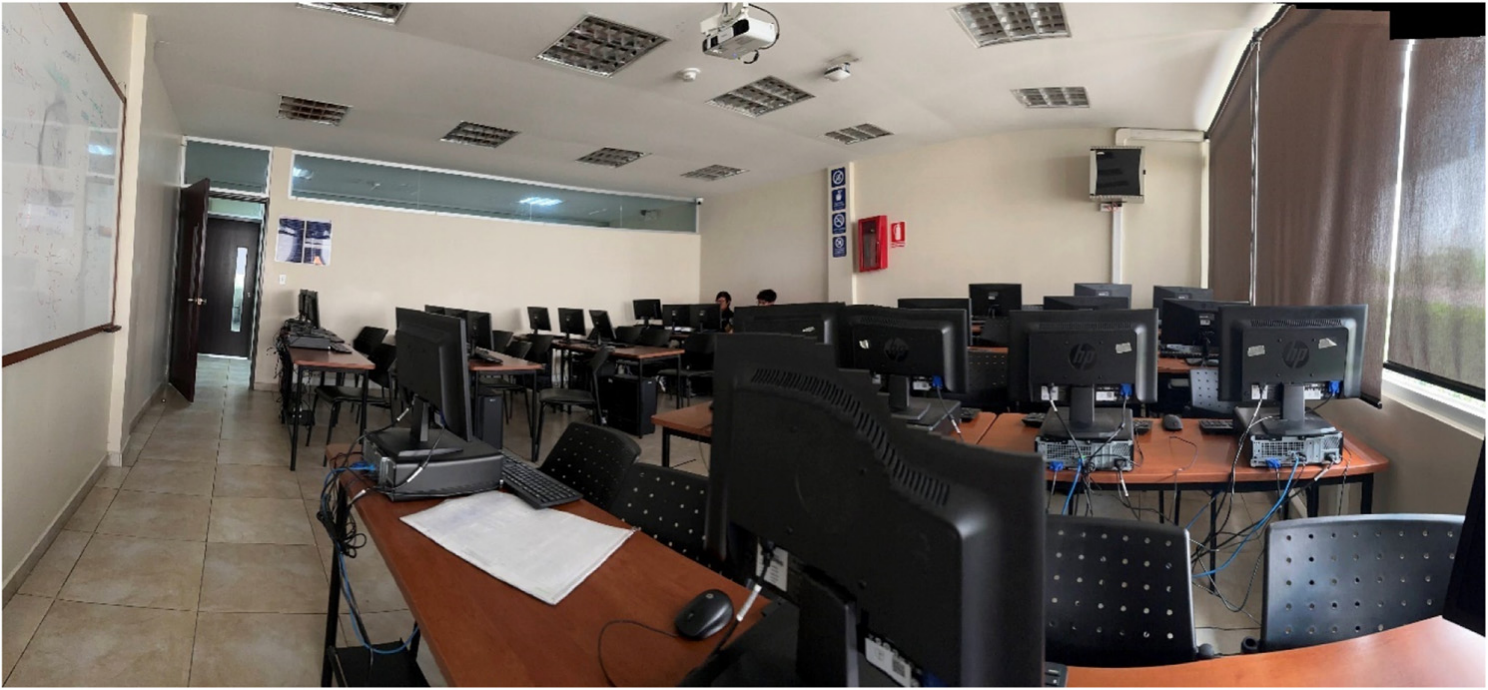
Computer laboratory setup during assessment session.

**Fig. 4:**
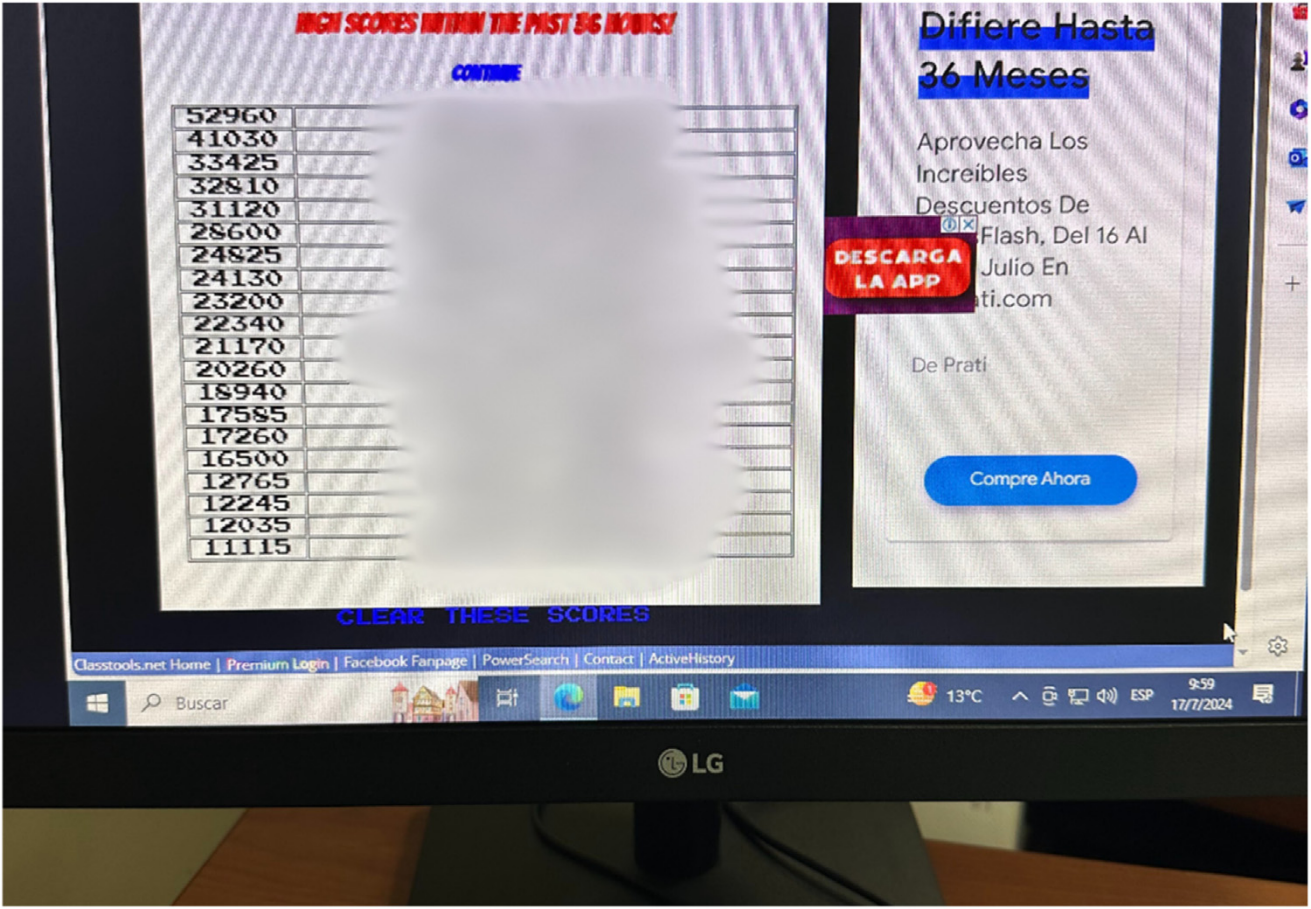
Leaderboard interface displaying achieved scores (names blurred for privacy).

Assessment sessions were conducted in computer laboratories at Universidad Indoamerica (Fig. 3). Each student was provided with an individual computer workstation for the two-hour evaluation period. The target score of 20,000 points was established as equivalent to the maximum grade (10/10), with scores scaled proportionally (score/2000). A leaderboard system tracked and displayed top scores to encourage engagement (Fig. 4).

In contrast to some research related to the topic of gamification [7], the whole process of replicating the evaluation methodology is described. The platform classtools.net has been used, which is freely available.

### 4.4 Data collection

**Fig. 5:**
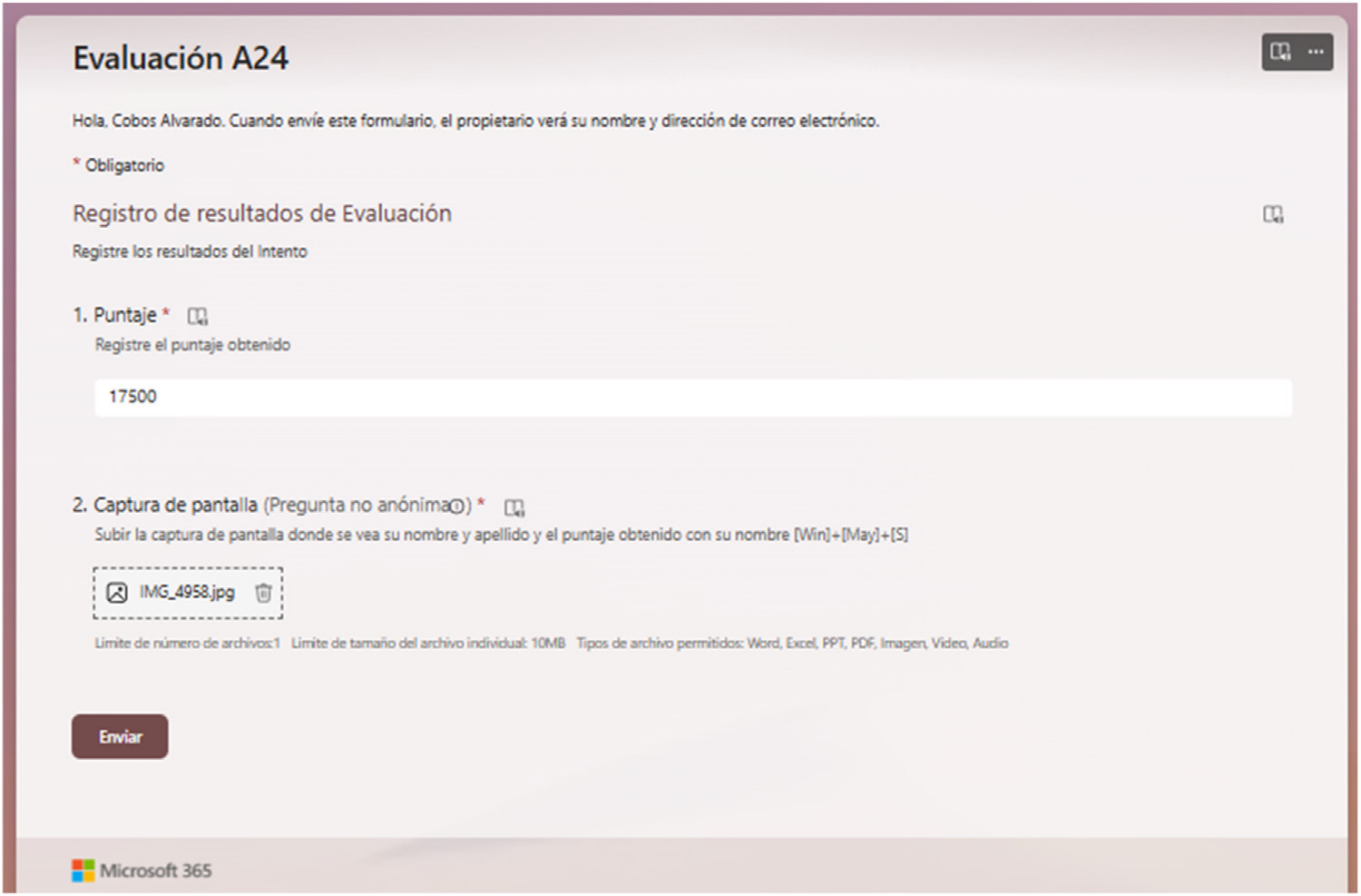
Microsoft Forms interface used for registering assessment attempts.

The data collection process was implemented through a structured validation system. Each attempt was documented through a Microsoft Forms interface (Fig. 5) where students:

• Authenticated using their institutional email account
• Submitted their achieved score
• Uploaded a screenshot of their attempt as evidence

The authentication system through institutional email accounts automatically provides student demographic data and program information, eliminating the need for manual input of these details.

To ensure data integrity, a two-step validation was employed. This approach aligns with contemporary frameworks, which recommend combining multiple sources of verification to ensure the accuracy of the results [10]. In line with these methodologies, this study implemented an institutional authentication system along with cross-validations between screenshots and the game's leaderboard system, minimizing possible inconsistencies.

**Fig. 6:**
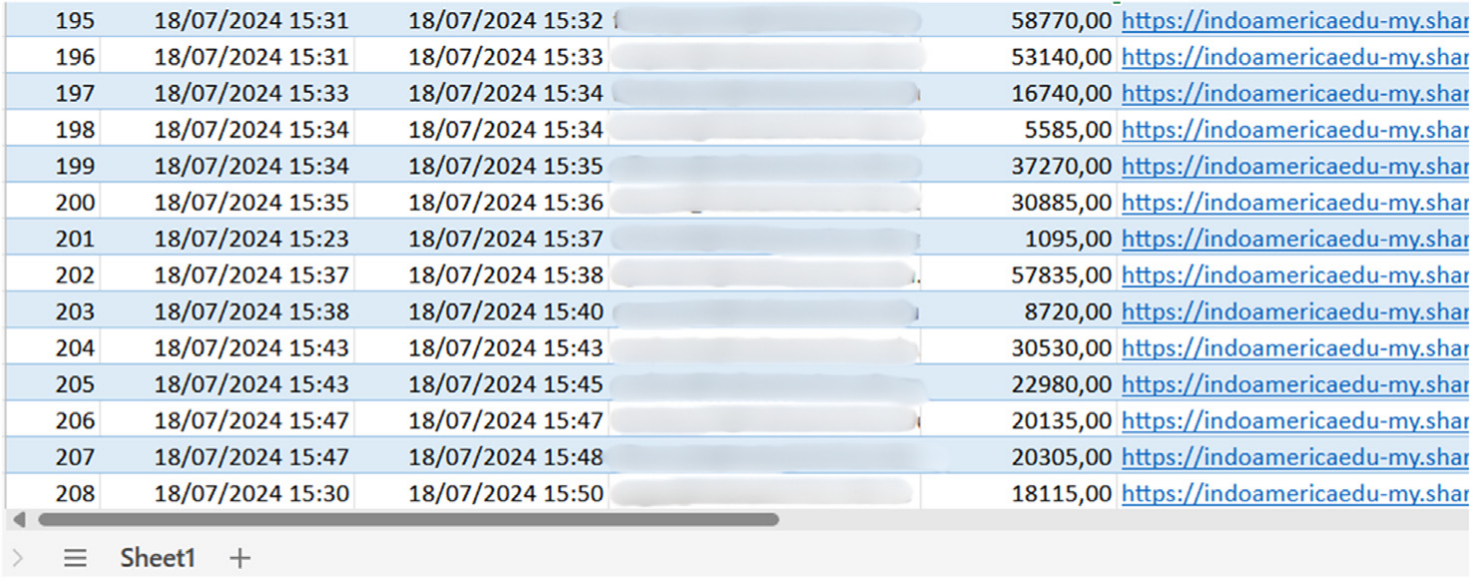
Sample of the structured Excel spreadsheet.

All data were automatically compiled into a structured Excel spreadsheet (Fig. 6), recording:

• Attempt timestamp (start and end time)
• Student institutional identification
• Score achieved
• Evidence link to the screenshot

**Fig. 7:**
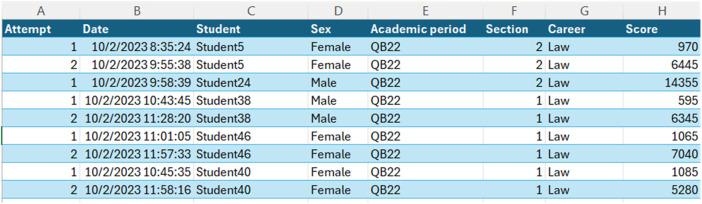
Data validated, integrated, and published.

This comprehensive experimental setup, combining the game mechanics with systematic data collection and validation processes, enabled the gathering of 1418 assessment attempts across four academic periods. The standardized implementation in a controlled laboratory environment, coupled with automated data collection and verification systems, ensured consistency and reliability in the assessment process. The column originally labeled as 'Login ID' has been replaced with 'Student' containing anonymized identifiers to ensure compliance with ethical standards for data privacy. Fig. 7 shows an example of the data reviewed, integrated and published.

## Limitations

The game platform presents a technical limitation in the leaderboard system, which cannot display scores exceeding 60,000 points. While this affected only three cases out of the entire dataset, these attempts were properly documented through the students' form submissions and screenshot evidence.

Some data collection inconsistencies were observed:

• A small number of low-scoring attempts were not registered by students who considered these scores insignificant
• Minor formatting inconsistencies occurred in score reporting, where some students used different thousand separators (comma or period). However, these formatting issues were easily standardized during the grade recording process.

Additionally, it is not possible to track which specific questions were presented in each game attempt or whether they were answered correctly or incorrectly. This limitation is due to the randomization mechanism of the question selection process, which does not log detailed question-level information. As a result, analyses related to question difficulty or response patterns could not be performed.

## Ethics Statement

The author confirms that this data collection was conducted as part of regular academic activities at Universidad Indoamerica. The assessment process was carried out in accordance with institutional policies for academic evaluation and data protection. Students participated in these evaluations as part of their standard course requirements in digital culture and computing courses. All data collection was conducted through official institutional platforms (Microsoft Forms) and authenticated institutional email accounts. The reported data contain only standard academic information (institutional id, gender, program, scores) that is routinely collected for educational purposes. No additional personal information was collected beyond what is standard for academic records, and data presentation has been appropriately anonymized where necessary.

## CRedit Author Statement

**Miguel Cobos:** Conceptualization, Methodology, Investigation, Resources, Data curation, Writing - Original Draft, Writing - Review & Editing.

## Data Availability

Mendeley DataResults of the evaluation with video games (Original data).
